# Endocrine Aspects of Environmental “Obesogen” Pollutants

**DOI:** 10.3390/ijerph13080765

**Published:** 2016-07-28

**Authors:** Francesca Nappi, Luigi Barrea, Carolina Di Somma, Maria Cristina Savanelli, Giovanna Muscogiuri, Francesco Orio, Silvia Savastano

**Affiliations:** 1 I.O.S. & COLEMAN Srl; 80100 Naples, Italy; luigi.barrea@unina.it (L.B.); cristysav@hotmail.com (M.C.S.) giovanna.muscogiuri@gmail.com (G.M.); 2 IRCCS SDN, Napoli Via Gianturco 113, 80143 Naples, Italy; cdisomma@unina.it; 3 Department of Sports Science and Wellness, “Parthenope” University of Naples, 80133 Naples, Italy; francescoorio@virgilio.it; 4 Dipartimento di Medicina Clinica e Chirurgia, Unit of Endocrinology, Federico II University Medical School of Naples, Via Sergio Pansini 5, 80131 Naples, Italy; sisavast@unina.it

**Keywords:** endocrine-disrupting chemicals, obesity, inflammation, obesogenic environment

## Abstract

Growing evidence suggests the causal link between the endocrine-disrupting chemicals (EDCs) and the global obesity epidemics, in the context in the so-called “obesogenic environment”. Dietary intake of contaminated foods and water, especially in association with unhealthy eating pattern, and inhalation of airborne pollutants represent the major sources of human exposure to EDCs. This is of particular concern in view of the potential impact of obesity on chronic non-transmissible diseases, such as type 2 diabetes, cardiovascular disease, and hormone-sensitive cancers. The key concept is the identification of adipose tissue not only as a preferential site of storage of EDCs, but also as an endocrine organ and, as such, susceptible to endocrine disruption. The timing of exposure to EDCs is critical to the outcome of that exposure, with early lifetime exposures (e.g., fetal or early postnatal) particularly detrimental because of their permanent effects on obesity later in life. Despite that the mechanisms operating in EDCs effects might vary enormously, this minireview is aimed to provide a general overview on the possible association between the pandemics of obesity and EDCs, briefly describing the endocrine mechanisms linking EDCs exposure and latent onset of obesity.

## 1. Introduction

Global obesity epidemics is most likely due to the interactions among heterogeneous causes, that include dysregulation of endocrine and metabolic systems, genetic predisposition, and environmental factors, in the context of the so-called “obesogenic environment” [1].

Although it has been estimated that the heritability of obesity range goes from 30% to 70%, the relative weight of genetic factors and environmental influences might be difficult to disentangle [2]. Epigenetics is a potential link between environmental exposures and gene activity, and both obligatory and facilitated epigenetic variations could account for the missing heritability of obesity [3]. Novel molecular approaches evaluating the phenotypic discordance in monozygotic twins, such as genome-wide methylation assays, point out that epigenetic changes induced by lifestyle are likely operating distinctly for each individual in the pathogenesis of obesity and related comorbidities [4].

Among the “obesogenic” environmental factors, a growing body of evidence supports that the exposure to certain environmental pollutants can contribute to the pandemics of obesity also independently of unhealthy diet and physical inactivity [5], especially in wealthy industrialized countries and in urban settings with elevated concentrations of primary air pollutants [6]. This is of particular concern in view of the potential impact of obesity on chronic non-transmissible diseases, such as type 2 diabetes, cardiovascular disease, and hormone-sensitive cancers. In both animal models and humans, the majority of environmental pollutants work by perturbing the endocrine systems and altering hormone action as endocrine-disrupting chemicals (EDCs) [5,7]. Increasing knowledge on the causal link between health effects and EDCs represents a great challenge for health care systems considering that, according to currently available literature, the socio-economic burden of EDC-related diseases for the countries of the Eurozone ranges between 46 and 288 billion €/year [8].

The aim of this minireview is to provide a general overview on the possible association between the pandemics of obesity and most common environmental pollutants acting as EDCs, briefly describing the endocrine mechanisms linking EDCs exposure and latent onset of obesity.

## 2. Endocrine-Disrupting Chemicals (EDCs)

EDCs are described as “exogenous chemicals or mixtures of chemicals that alter function(s) of the endocrine system and consequently cause adverse health effects in an intact organism, or its progeny, or (sub)populations” (World Health Organization, WHO). EDCs are ubiquitous in the environment, including atmosphere, sediments, soils, water, and are derived both from industrial chemicals, such as bisphenol A and phthalates used as plastic packaging components and other consumer products, from agricultural sources, such as pesticides, fungicides, insecticides, or herbicides, or can occur naturally, such as phytoestrogens. Human pollutant exposure is predominantly through the consumption of contaminated tap water and food, mainly meat, fish, and dairy products, especially in association with unhealthy eating pattern, or by dermal exposure due to direct contact of the skin with the EDCs or from the ambient air, and by inhalation of volatile contaminants and airborne fine and ultrafine particulate matter (PM) often generated through widely-used thermal processes, such as the combustion of fuels or the thermal decomposition of waste [9].

Based on the World Health Organization/International Programme on Chemical Safety 2002 definition of EDCs [9], the vast majority of the POPs currently listed in the Stockholm Convention are EDCs (i.e., those chemicals that have potential to interact with the hormone systems, but the adversity of such interaction is yet to be studied or recognized). As many EDCs are lipid soluble, they easily accumulate in human tissues throughout a life span, mainly in adipose tissue and in overweight/obese populations [9]. EDCs can interfere with the endocrine system at multiple levels, by influencing hormone synthesis, release, transport, metabolism and excretion [7]. In particular, EDCs act by agonizing or antagonizing the effects of hormones through mechanisms dependent or independent of nuclear receptors, resulting in the transcription of target genes involved in homeostasis and developmental processes. A major issue in the study of EDCs is the apparent complexity of their mechanisms of action, which may occur through multiple signaling pathways and targets. In addition, the exposure to different levels of EDCs produces diverse phenotypes, thereby resulting in nonmonotonic dose-response curves [10]. Notably, the complexity is further increased as animals and humans are exposed to complex mixtures of EDCs instead of single compounds. Thus, the mechanisms operating in EDCs effects might vary enormously.

Adverse effects of about 800 chemicals with EDCs properties that are used in daily life have been shown in both humans and wildlife [9]. The endocrine disrupting effects in relation to different systems, organs and sources of exposure have been examined in a recent review, including both experimental and epidemiological data [11]. A key role for EDCs has been proposed in the pathogenesis of early puberty, infertility, altered quality of sperm, and reproductive disorders later in life in both animal and human studies [11]. Altered thyroid and adrenal hormones biosynthesis and functions have been also included among commonly occurring EDCs adverse effects [11]. In addition, a growing body of evidence indicates a pivotal role for EDCs in promoting the obesity epidemics [12].

## 3. “Obesogenic” EDCs from the Endocrinologic Point of View

The global increase in the production of EDCs parallels the worldwide increase in obesity prevalence, lending support to the “obesogen” hypothesis [7]. A role for EDCs in the etiology of obesity was initially postulated in the early 2000s when Baillie-Hamilton reported that the global obesity epidemic coincided with the marked increase of industrial chemicals in the environment over the past 40 years [13]. Since the Baille-Hamilton review, an increasing number of experimental and clinical studies support the key role for exposure to EDCs in the obesity epidemics [14,15,16]. The term “obesogens” was coined by Grun and Blumberg as “xenobiotic chemicals that can disrupt the normal developmental and homeostatic controls over adipogenesis and/or energy balance” [17]. According to the “obesogen hypothesis”, the prenatal or early-life exposure to certain EDCs predisposes some individuals to gain excessive adipose tissue [7]. The key concept is the identification of adipose tissue not only as a preferential site of storage of EDCs, but also as an endocrine organ and, as such, susceptible to endocrine disruption, especially in critical windows of development, such as pre and early postnatal life, or puberty. Of interest, while the weight gain in adults is predominantly due to changes in size of pre-existing adipocytes in most adipose depots, the increased adipogenesis during early development permanently establishes an elevated adipocyte number in adulthood. The inability of adipose tissue to further expand in order to tackle the chronic positive balance between food energy intake and expenditure, is responsible for the development of a dysfunctional adipose tissue that contributes to obesity and obesity-associated metabolic complications [18].

In general, the environmental exposure to specific EDCs, identified as “obesogenic and/or diabetogenic”, induces obesity by disrupting the homeostatic control of adipogenesis and/or energy balance [19]. There are multiple mechanisms involved in EDC-induced obesity. “Obesogenic” EDCs interfere with different endocrine-regulated metabolic processes, including lipid and glucose metabolism and insulin signaling pathway [11,20]. In addition, “obesogenic” EDCs promote adipogenesis in cellular models, promote either adipogenesis and obesity in animal models as well as in humans [5], and influence the neuroendocrine control of appetite and satiety in experimental animals [9]. Furthermore, EDCs interfere with pro-inflammatory mechanisms by activating oxidative stress-sensitive transcription factors, such as nuclear factor kappa-light-chain-enhancer of activated B cells (NFκB), and inducing cytokines, chemokines, and adhesion molecules in the vascular endothelium. Low grade chronic inflammation can be considered a strong basis for the metabolic alterations of obesity epidemics, such as visceral adiposity and insulin resistance [21]. Through these pro-inflammatory mechanisms, EDCs are correlated with an increased risk of non-communicable or chronic diseases, such as atherosclerosis, type 2 diabetes, obesity, nonalcoholic fatty liver (NAFLD), the hepatic expression of the metabolic syndrome, cardiovascular disease, and hormone-sensitive cancers later in life [11,22,23,24]. In this, EDCs could represent a model of the interaction between environmental and genetic factors involved in the development of obesity/diabetes/metabolic syndrome, although the metabolic consequences might vary with gender, and dose and time of EDCs exposure [5]. Of interest, the adipose tissue expansion might play a dual role of promoting metabolic disorders and type 2 diabetes on the one side, but providing a relatively safe place to store EDCs on the other side.

Epigenetic variations and shift in gut microbiota profile are sensible targets for EDCs “obesogen” effects. Possible mechanisms by which early life EDC exposure can affect epigenetic programming of obesity are binding, activation or inhibition of nuclear receptors and other transcription factors that regulate the expression of the target genes [25]. The timing of exposure to EDCs is critical to the outcome of that exposure, with early lifetime exposures (e.g., fetal or early postnatal) particularly detrimental because of their permanent effects [21]. Data on both animal and human data clearly indicates that exposure to “obesogenic” EDCs during critical periods in the early life, such as during organogenesis and around puberty, induces epigenetic, transgenerational modifications, i.e., heritable changes of gene expression without accompanying changes in the DNA sequence manifested across multiple generations [26]. The epigenetic, transgenerational modifications induced by EDCs include expression of noncoding RNAs, alterations in chromatin structure, and transcriptional activity resulting from changes in DNA and histone methylation. Low levels of DNA methylation at gene promoters might generate active adipogenic genes, thereby permanently increasing adipocyte number and favoring central fat deposition in the presence of energy imbalance. The hyperactivity of adipogenic genes fosters to create an altered metabolic set point, thereby influencing latent effects on the risk of obesity and obesity-related outcomes, and likely accounting also for the very common issue of weight regain after weight loss [27]. Of interest, novel molecular approaches evaluating the phenotypic discordance in monozygotic twins, such as genome-wide methylation assays, point out that epigenetic changes induced by lifestyle are likely operating distinctly during adolescence/adult life for each individual in the pathogenesis of obesity and related comorbidities [4]. Accordingly, maternal weight loss is associated with changes in gene methylation/expression of different inflammatory pathways in the offspring and reduced inflammatory marker levels [28].

**Figure 1:**
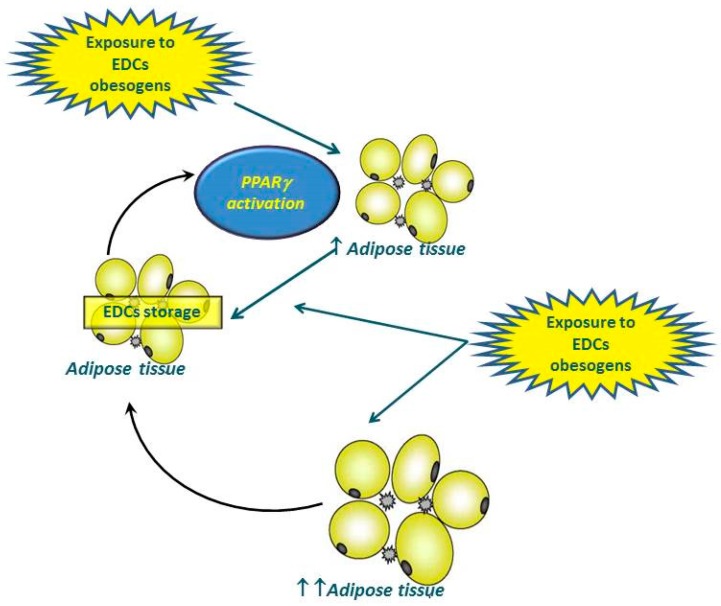
EDCs are known to activate PPAR-γ, which leads to weight gain in vivo preferentially committing the mesenchymal stem cells toward differentiation into adipocytes regulating the relative expression of PPARγ-induced genes. As the vast majority of EDCs are lipophilic, these compounds might be easily accumulated in the adipose tissue over the years. Thus, a continuous spiral is created, with the increasing burden of EDCs stored in the body fat along with the EDC-induced accrual of adipose tissue. ***EDCs***, endocrine-disrupting chemicals; ***PPAR-γ***, Peroxisome Proliferator-Activated Receptor-γ.

The most common epigenetic variations in obesity in humans have been evaluated by a recent meta-analysis [29]. In particular, EDCs act as ligands for the Peroxisome Proliferator-Activated Receptors-γ (PPARγ) and their binding is associated with DNA methylation variation of PPARγ or PPARγ target genes. PPARγ is a pivotal molecule in the regulation of adipogenesis highly expressed in adipose tissue, where it regulates not only the differentiation into adipocytes, but also the accumulation of triglycerides, glucose metabolism, and insulin sensitivity in mature adipocytes [30,31,32]. “Obesogenic” EDCs can potentially promote adipose tissue accrual during early development preferentially committing the mesenchymal stem cells toward differentiation into adipocytes in a process dependent by binding to PPARγ, with regulation of the relative expression of PPARγ-induced genes. As the vast majority of EDCs are lipophilic, it therefore flows that these compounds might easily accumulate in the adipose tissue over the years. Thus, a continuous spiral is created, with the increased burden of EDCs stored in the body fat along with the EDC-induced accrual of adipose tissue [19] (Figure 1). Further DNA methylation variations associated with exposure to EDCs occur in the promoter of the retinoid X receptor α gene (RXRα), a nuclear receptor reported to explain up to 26% of the variation in childhood adiposity. RXRα exerts a pivotal role in adipogenesis by forming a heterodimer with PPARγ, and in PPARγ coactivator-1 (PGC-1), a tissue-specific transcriptional coactivator associated with weight loss, obesity, and risk for type 2 diabetes mellitus [29]. In addition to binding to PPARγ, EDCs promote preadipocyte differentiation through different regulatory pathways, including the agonistic effects on estrogen (ER), glucocorticoid, and aryl-hydrocarbon receptors (AhR) [7]. Other possible mechanisms of EDCs action evidenced in experimental models include: direct effects on insulin signaling with decreased expression of insulin-dependent genes involved in lipid homeostasis in pre-adipocyte 3T3-L1cell line, activation of genes related to the inflammatory pathway in human adipocytes likely through the AhR, increased mRNA expression, as well as the enzymatic activity of 11β-hydroxysteroid dehydrogenase type 1, known to induce adipogenesis in humans by converting cortisone to cortisol in adipose tissue.

Both the host gastrointestinal tract and the commensal gut microbiota are likely to be exposed to and modulated by EDCs through the diet. A number of studies suggest that the interactions between gut microbiota and environmental toxicants may contribute in part to the development of obesity and diabetes [33,34]. In particular, the AhR, a receptor highly expressed by intestinal intraepithelial lymphocytes and actively controlling immune homeostasis in the gut, is bound and activated by a variety of polychlorinated biphenyls (PCBs). Recent evidence indicates that gut microbiota, which can be modulated by the AhR [35], may play a pivotal role in the “obesogen” effects of these compounds. In animal experimental studies, EDCs, in particular 2,3,7,8 tetrachlorodibenzofuran, alter the composition of the gut microbiota after its binding to the AhR [34]. Changes in gut microbiota profile are associated with the alteration of their inherent metabolic activity, with significantly increased levels of bile acids and short-chain fatty acids, altered liver function, increased intestinal inflammation, and inhibited signaling of the farnesoid X receptor, a key regulator of fat and glucose metabolism [36].

Below it is provided a more detailed description concerning the EDCs more commonly associated with obesity.

## 4. Diethylstilbestrol

Diethylstilbestrol (DES) is a non-steroidal estrogen that was initially used to prevent adverse pregnancy outcomes from the late 1940s to 1971 and commonly used as a growth promoter in animal production [37]. The mechanism of DES toxicity includes its interference with the reproductive system and association with female reproductive tract cancers, in both exposed women and their offsprings [38].

Animal studies suggested that, although multiple pathways might be involved in the programming for obesity, DES exposure during prenatal or perinatal period was linked to weight and body fat increase, persisting in adulthood [39]. It was hypothesized that DES exposure works on the genetic programming of adypocites and on their distribution by interacting with ER and different genes and transcriptional factors, including gene implicated in altered adipocyte differentiation and function (*Hoxa5*, *Gpc4*, and *Tbx15*), as well as fat cell distribution (*Thbd*, *Nr2f1*, and *Sfrp2*) [40]. In addition, high levels of markers of adiposity, such as leptin, and proinflammatory cytokines, such as interleukin 6 (IL-6), were found in mice models in the early phase of exposure, suggesting that these could constitute early markers for the development of metabolic syndrome and obesity related diseases later in life. Alterations in glucose metabolism along with a high prevalence of pancreatic β-cell hyperplasia were also found in DES exposed mice [39]. Data from the National Cancer Institute DES Follow up Study on prenatally DES-exposed women supported the association between DES and adult obesity in humans [37]. In particular, the adjusted risk ratio for DES and obesity among 2871 prenatally exposed compared to 1352 unexposed women aged 23–52 years at baseline in 1994 was 1.09 (CI: 0.97–1.22), thus suggesting that prenatal DES exposure might be associated with a small increase in adult obesity.

## 5. Bisphenol A

Bisphenol-A (BPA), a component of polycarbonate plastics and resins, is one of the highest-volume chemicals produced worldwide. BPA has largely been found in the production of food and beverages into which BPA leaches from polycarbonate containers, including reusable bottles baby bottles, toys. However, BPA is also commonly found in thermal paper and also dental composites. Human BPA exposure can occur through ingestion, inhalation, or dermal absorption from contaminated materials. Foods and water can be contaminated by BPA monomers as a result of the heating or either acidic or basic conditions during storage. BPA has been detected in 93% of urine samples of the general population in the United States [24]. Due to its nearly ubiquitous environmental contamination, BPA concentration in human serum ranges from 0.2 to 1.6 ng/mL [25], but BPA has been found also in amniotic fluid, neonatal blood, placenta, cord blood, and human breast milk [41,42,43].

It is known that BPA is a ER panagonist, and the interaction with ERs influences the expression of estrogen-responsive genes [44,45,46], interfering with reproductive function [47,48]. In addition to its well documented actions on the reproductive system, BPA exerts a wide variety of metabolic effects. Although the pancreatic islets and adipocytes are not considered classical targets of estrogen, both express functional ERs [49]. Consequently, BPA exposure has been demonstrated to have a key role in the pathogenesis of multiple metabolic disorders, promoting insulin resistance, disruption of pancreatic β-cell function, hepatotoxicity, and obesity [50]. Data from the 2003/04 and 2005/06 National Health and Nutrition Examination Surveys (NHANES) evidenced that urinary BPA concentration in adult population was positively associated with general and central obesity, especially in men [51].

BPA is a lipophilic molecule that accumulates in adipose tissue, where interferes in the mechanisms of adipose tissue differentiation. in vitro experiments revealed that in both 3T3-L1 cells and human adipose stromal/stem cells the presence of BPA promoted an increase in triglyceride content and adipogenesis, inhibited the adiponectin release [50], and increased the release of proinflammatory cytokines, such as IL-6 and IFN-γ, by activation of JNK, STAT3 and NFkB pathways [52], while in a hepatoma cell line it increased triglyceride content and lipid accumulation along with the decrease in expression of some genes involved in lipid oxidation [53]. More recently, in 3T3-L1 pre-adipocytes BPA increased the expression of PPAR-γ, Fatty Acid Binding Protein 4/Adipocyte Protein 2 (FABP4/AP2) and CCAAT/enhancer binding protein (C/EBPα), while in mature adipocytes BPA increased lipid accumulation and reduced insulin-stimulated glucose utilization [54]. The adipogenic effect of BPA has been proposed to be mediated by an ER-dependent mechanism, along with the enhanced expression of DLK (leucine zipper-bearing kinase), insulin-like growth factor-1 (IGF-1), C/EBPα, or PPARγ among other factors [14,55]. Furthermore, BPA has been reported to promote the adipogenesis by increasing expression and enzyme activity of 11β-hydroxysteroid dehydrogenase type 1 [56], the thyroid receptor/retinoic X receptor or mammalian target of rapamycin signaling pathways [57]. Previous in vivo studies have demonstrated that the prenatal BPA exposure is linked to DNA methylation variation of Insulin-like Growth Factor 2 (IGF-2) and H19, two reciprocally expressed imprinted genes located on chromosome 11p15.5 that play a major role in fetal and placental growth [29].

**Figure 2:**
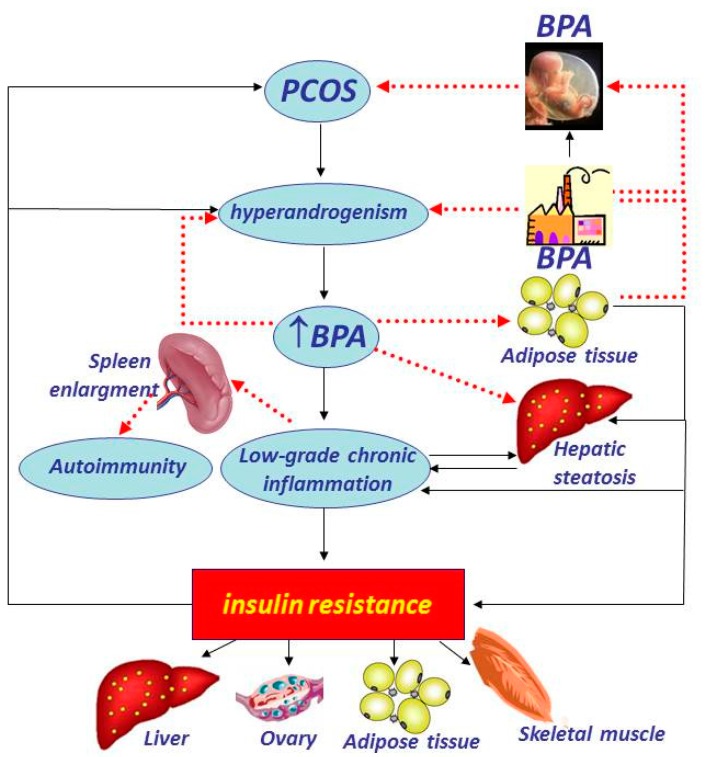
A possible pathway involving BPA, liver-spleen axis, hyperandrogenism, and low-grade inflammation in pathogenesis of the polycystic ovary syndrome taking into account the suggested role of BPA as EDC for the “environmental obesogen hypothesis”, and considering the possible relationship between in utero exposure to BPA and PCOS pathogenesis. Besides a direct hepatotoxic and adipogenetic effect, BPA could act as pro-inflammatory primer, via macrophage activation and pro-inflammatory cytokine hypersecretion, being spleen enlargement as marker of this process, with a possible link with immune/autoimmune derangement in women with PCOS. PCOS exposure to low-chronic BPA doses might initiate or exacerbates obesity and IR, while in turn, BPA influences androgen metabolism. ***BPA***, Bisphenol A; ***EDCs***, endocrine-disrupting chemicals; ***PCOS***, polycystic ovary syndrome; ***IR***, insulin resistance.

Finally, low grade chronic inflammation and adipocyte dysfunction have been also proposed as the causal link between BPA and obesity in different clinical settings, including subjects with visceral obesity [58] and women with polycystic ovary syndrome (PCOS) [59]. The possible relationships between BPA and PCOS pathogenesis, involving in utero exposure to BPA, liver-spleen axis, hyperandrogenism, and low-grade inflammation, are depicted in Figure 2. In particular, besides a direct hepatotoxic and adipogenetic effect [50], BPA could act as pro-inflammatory primer, via macrophage activation and pro-inflammatory cytokine hypersecretion, with a possible link with immune/autoimmune derangement in women with PCOS. PCOS exposure to low-chronic BPA doses might initiate or exacerbates obesity and insulin resistance, while in turn, BPA influences androgen metabolism.

## 6. Phytoestrogens

Phytoestrogens are naturally-occurring plant compounds found in a wide variety of foods, most notably soy, and commonly known as “dietary estrogens”. Indeed, exposure to phytoestrogens is mainly due to the increased consumption of soy products and soy supplements in the effort to implement healthier eating lifestyles. Soy proteins are used as a meat substitute in hotdogs, hamburgers, sausages and other meat products or are widely available as dietary supplements to enrich energy bars, sports drinks, infant formulas, cereals, imitation dairy products, and ice cream. The health benefits frequently attributed to phytoestrogens include a lowered risk of osteoporosis, heart disease, lipid disorders, breast cancer, and menopausal symptoms [60]. However, because of their chemical analogy with 17-β-estradiol, phytoestrogens are able to induce estrogenic or anti-estrogenic effects, interacting with ERs. Therefore, phytoestrogens are also considered EDCs, indicating that they have the potential to cause adverse health effects as well [61]. Besides their primary role as EDCs on reproductive outcomes, phytoestrogens are also supposed to interact with the normal adipose tissue regulation and proliferation, mainly in the early period of development. Genistein, the predominant isoflavone in soybean, has been proposed as a promising compound for the treatment of metabolic disorders due to its anti-oxidant and anti-inflammatory effects [60]. However, at the low dosage commonly found in Western and Eastern diet, genistein seems to be responsible of changes in the expression of metabolic and adipogenic regulators, such as PPAR-γ and others, thus promoting fat accumulation in adipose tissue, especially in males [62]. Recent experimental data supported that genistein dysregulates the body composition, in a dose-dependent and gender-specific manner, disrupting and reprogramming the signals dictating adipose tissue expansion, likely throughout the early-life epigenetic regulation of *Wnt10b*, a further key adipogenic gene in adipose tissue [15].

## 7. Phthalates

Phthalates are diesters of phthalic acid and are used in the production of plastics to improve the quality of these products, such as polyvinyl chloride (PVC), and are also commonly found in many consumer products, such as food and beverage packaging, soaps, shampoos, cosmetics, and hairsprays. Phthalates are released into the environment from PVC and plastic materials, so human exposure can easily occur through dermal absorption from contaminated materials or inhalation or ingestion [63]. In particular, children can be easily exposed to the effects of phthalates released in food or beverages containers, plastic toys or other plastics.

Researches in mice models show that phthalates metabolites could be responsible for increasing adipogenesis and insulin resistance by activating PPAR-γ receptors expression in the period of adipocytes differentiation [64]. Among results of human studies investigating the effects of phthalate exposure on obesity, data from the National Health and Nutrition Examination Survey (NHANES) 1999–2002 evidenced a positive association between urinary excretion of phthalates metabolites and waist circumference in both male and female adults was found [65], while more recent studies from NHANES 2007–2010 found that phthalates were associated with higher odds for obesity in both adults and children of both gender [66].

A further potential mechanism linking phthalates to obesity might be provided by the anti-androgenic effects of these compounds. Of interest, testosterone is a key hormone in the pathology of metabolic diseases and obesity, and a low androgenic activity has been related with the development of overweight and obesity [67]. The anti-androgenic effects might be mediated via the inhibition of Leydig cell steroidogenesis at different ages of fetal development [68] or by indirectly interacting with the normal activity of androgen receptors, via the PPAR-α expressed in gonads [69].

## 8. Organochlorine and Organophosphate Pesticides

In the general population, the dietary intake of pesticides is the most important source of EDC exposure [70]. Organochlorine pesticides (OCPs) are chlorinated hydrocarbons used from the 1940s through the 1960s and then banned in the United States and many other countries according to the Stockholm Convention on Persistent Organic Pollutants in 2001. Unfortunately, some of these compounds are still registered for use and are detected in tap water, posing a serious risk to worldwide human health and the environment. Organophoshates (OPPs), esters of phosphoric acid, have often been selected to replace persistent OCPs and represent up to 50% of all the insecticide use worldwide [70]. The accidental inhalation or ingestion of these compounds in fish, dairy products, and other fatty foods that are contaminated, represent the most common way of human exposure. After entering into the food chain, these compounds tend to persist in the environment and accumulate in fat mass of mammals due to their high lipophilicity, causing disrupting effects on endocrine, immune, and reproductive systems [20].

Exposure to OCPs has been linked to metabolic disorders, such as obesity and type 2 diabetes. Inflammation, as a known mechanism accompanying insulin resistance, has also been shown to arise in insulin target tissues exposed to OCPs. In recent studies, selected OCPs are recognized to predict insulin resistance, higher body weight and altered lipidic profile in exposed populations [71]. Several hypotheses to explain how these chemicals could give metabolic dysregulation have been analyzed, including interference on PPARγ expression, production of inflammatory cytokines, such as Tumor Necrosis Factor-α (TNF-α), and alterations in steroid metabolism with anti-androgenic effect [72]. Very recently, OCPs such as p,p′-dichlorodiphenyldichloroethylene (DDE), have been indicated as responsible of enhancing adipogenesis and intracellular lipid accumulation in 3T3-L1 cells through up-regulation of molecular targets responsible for lipid storage, including fatty acid binding protein 4 and Sterol regulatory element-binding protein-1c [73].

A strict link has been reported between the early-life OPPs exposure and the subsequent emergence of hyperinsulinemia and hyperlipidemia, depicting an overall pattern essentially resembling prediabetes, through the pathway synthesizing cyclic AMP controlled by adenylyl cyclase, the common site for disruption by OPPs [70]. In addition, recent evidence indicated that dietary chronic exposure to chlorpyrifos, the most used OPPs worldwide, increased food intake and promotes weight gain in mice with Apolipoprotein E3 isoform, the most common in humans, unraveling relevant interactions between toxic exposure to chlorpyrifos and genetic predisposition [74].

## 9. Polychlorinated Bisphenols

PCBs are a class of organic chemicals widely used, mainly in electrical equipment. Although PCBs were banned at the end of the 1970s in many countries because of environmental toxicity, these compounds still remain present in the environment due to their high stability and represent one of the key constituents of POPs all over the world [75]. On the basis of their biochemical properties, some PCBs are classified as dioxin-like compounds and exert many effects after their the binding to the AhR. In addition to their neurotoxicity, some PCBs are identified as EDCs because of their estrogenic and antiandrogenic effects [76] and interference with thyroid metabolism [77]. As documented by prospective epidemiologic studies, PCBs act as “obesogens” when exposure occurs in fetal period, during the phases of adypocites differentiation [78,79]. PBCs exposure is associated with the development of the metabolic syndrome and childhood obesity, with a clear gender difference as the associations were stronger in girls compared to boys [80].

## 10. Perfluoroalkyl Substances

Perfluoroalkyl substances (PFASs) are ubiquitous non-organochlorine POPs, widely used in industrial applications and detectable in the blood of human populations worldwide. Similarly to other POPs, they are able to persist in the environment, and to accumulate in human tissues, with adverse effects on exposed individuals [81]. The contamination can occur through the contact of food, drinking water, or inhaling dust [82]. Some recent researches reported on the association between PFASs exposure in prenatal life and the development of overweight in childhood [83,84]. Different targets were identified for different groups of PFASs. Indeed, PFAS are known to activate PPARα, a receptor involved in regulating gene expression related to lipid and glucose metabolism, but PPARα-dependent or independent mechanisms vary widely across PFASs [85]. In particular, plasma concentrations of PFASs were positively associated with both total and HDL cholesterol in pregnant women, where higher total cholesterol is associated with elevated risk of preeclampsia [86].

## 11. Inhaled Pollutants

In 2004, the first American Heart Association scientific statement on “Air Pollution and Cardiovascular Disease” concluded that exposure to PM air pollution contributes to cardiovascular morbidity and mortality [87]. The association between obesity and the cardiovascular health effects of fine particulate air pollution (PM_2.5_) has been extensively evaluated by a meta-analysis including three large prospective cohort studies and 14 panel studies with short-term follow-up [88]. Results of this meta-analysis suggested that obesity may modify the impact of PM_2.5_ on cardiovascular health, as obese people may be more susceptible to the cardiovascular health effects of ambient PM_2.5_, with a higher risk of cardiovascular mortality among obese subjects also after adjusting for a number of potential confounding factors. Endothelial dysfunction and reactive oxygen species generation via activation of alveolar macrophage and systemic vascular oxidases including the NAD(P)H, mitochondrial and xanthine oxidases, appear to mediate this risk [89]. In addition, PM_2.5_ exposure enhances the expression of proinflammatory cytokines, such as IL-6 and TNF-α [90]. Thus, mechanistically, inhaled pollution particles induce a local inflammatory response in the lung that is initiated by alveolar macrophages and airway epithelial cells. Subsequently, these systemic mediators translocate from the lung into the circulation eliciting the classic systemic inflammatory response, with production of acute phase proteins by the liver. The increase in circulating leukocytes, platelets, and proinflammatory/prothrombotic proteins could constitute the link for the development of obesity, insulin resistance, endothelial dysfunction and progression of atherosclerosis induced by PM_2.5_ exposure [91]. Accordingly, recent studies evaluated a role for environmental exposition to PM_2.5_ in the development of metabolic abnormalities typical of obesity, type 2 diabetes and metabolic syndrome in both animal models and in humans. In high-fat diet-fed rats, inhaled pollutants increased insulin resistance, visceral fat, and systemic and cellular inflammatory markers [16]. In humans, there is a strong association between long-term air pollution exposure and various components of metabolic syndrome, with subsequent cardio-metabolic effects [92,93].

Further mechanisms have been described for the association between of inhaled pollutants and obesity. Environmental pollutants resulting from incomplete combustion, such as polycyclic aromatic hydrocarbons (PAHs) and including benzo[a]pyrene, are characterized by the capacity to bind to DNA, forming PAH–DNA adducts. Thus, besides their well known carcinogenic effects, PAHs might act as “obesogens” by altered methylation of PPARγ or PPARγ target genes. Furthermore, although limited data exists on the potential role of inhaled pollutants on liver disease in the general population, PM_2.5_ has the potential to induce Kupffer cell cytokine secretion by the liver, thus representing a significant risk factor for NAFLD progression [94]. In addition, Thomson and colleagues [95] have recently reported that inhalation of particulate matter or ozone increases plasma levels of the hypothalamic-pituitary-adrenal (HPA) axis stress hormones in rats to limit the inflammatory signaling in the lungs. The chronic stimulation of glucocorticoids induces well-characterized effects on both peripheral metabolism and food intake [96]. On the one hand, glucocorticoids up-regulate glucagon and inhibit insulin action, resulting in increased gluconeogenesis, impaired peripheral glucose uptake, and disruption of insulin receptor signaling [97]. On the other hand, glucocorticoids stimulate visceral fat accumulation, protein breakdown, and eating behavior by facilitating orexigenic activity of NPY and AgRP neurons [98]. In addition, during and after exposure to acute or chronic stress, the activation of HPA axis inhibits a number of endocrine axes, including somatotropic, thyrotropic and gonadal axes, which are all known to exert relevant effects on body composition and weight gain [99,100]. In this context, it is tempting to speculate that activation of HPA axis as response to the systemic inflammation induced by inhaled pollutants might elicit similar complex endocrine perturbations. Thus, the persistent dysregulation of HPA axis might lead to the amplification of detrimental effects on body composition, thereby potentially affecting the susceptibility to obesity and non-transmissible chronic diseases during lifespan.

## 12. Conclusions

The data included in this brief review support a mechanistic endocrine link between the early environmental exposure to EDCs and the development of obesity and of obesity-related diseases later in life.

The adipocytes are endocrine cells producing and receiving endocrine signals from different endocrine tissues and, as such, susceptible to EDCs, especially in critical windows of development. “Obesogen” actions of EDCs should be considered parts of a very complex scenario resulting from multiple and bidirectional relationships between EDCs and genetic, epigenetic and environmental factors, including parental diet/exposure, lifestyle, dietary habits, gut microbiota. In addition, it should be considered that the “obesogen” mechanisms operating early in life might vary enormously later in life due the chronic exposure to EDCs throughout the entire life span of the exposed individuals.

The wide range of different EDC classes and modality of exposure, the need of highly selective and sensitive analytical methods for the measurement of EDCs and the evaluation of the biological effects in humans and wildlife tissues, and the relative scarcity of information on socio-economic cost estimates, make it challenging to adopt primary preventive measures for dealing with the “obesogen” effects linked to the environmental exposure to EDCs. Nevertheless, as growing evidence indicates that the exposure to EDCs might account for a relevant part of the incidence of obesity and related-conditions, then stronger controls on EDCs could generate better health and significant financial savings each year for health care services [101].

Additional studies including prospective intervention trials in controlled populations, especially considering the important confounding effect of diet, are mandatory to determine the relevance of some hot points in the link between EDCs and obesity, including critical windows of susceptibility for various target tissues, dose-response curves, and potential synergistic effects of mixtures of EDCs and unhealthy eating patterns.
